# Effects of restraint stress on the daily rhythm of hydrolysis of adenine nucleotides in rat serum

**DOI:** 10.1186/1740-3391-9-7

**Published:** 2011-07-28

**Authors:** Andressa Souza, Bernardo C Detanico, Liciane F Medeiros, Joanna R Rozisky, Wolnei Caumo, Maria Paz L Hidalgo, Ana Maria O Battastini, Iraci LS Torres

**Affiliations:** 1 Laboratório de Cronobiologia Experimental, Departamento de Farmacologia, Instituto de Ciências Básicas da Saúde, Universidade Federal do Rio Grande do Sul, Porto Alegre, RS, 90050-170, Brazil; 2 Departamento de Bioquímica, Instituto de Ciências Básicas da Saúde, Universidade Federal do Rio Grande do Sul, Porto Alegre, RS, 90035-003, Brazil; 3 Programa de Pós-Graduação em Medicina: Ciências Médicas, Faculdade de Medicina, Universidade Federal do Rio Grande do Sul, Rua Ramiro Barcelos, 2400, Porto Alegre, RS, 90035-003, Brazil; 4 Unidade de Experimentação Animal, Grupo de Pesquisa e Pós-Graduação do Hospital de Clínicas de Porto Alegre, Universidade Federal do Rio Grande do Sul, Porto Alegre, RS, 90035-003, Brazil

**Keywords:** Adenine nucleotides hydrolysis, Circadian rhythm, Rats, Restraint stress, Temporal pattern

## Abstract

**Background:**

Adenosine 5-triphosphate (ATP) and its breakdown products ADP and adenosine can act as extracellular messengers in a range of biological processes. Extracellular adenine nucleotides are metabolized by a number of enzymes including NTPDases and 5'-nucleotidase, which are considered to be the major regulators of purinergic signaling in the blood. Previous work by our group demonstrated that ATPase and ADPase activities in rat serum exhibit a 24-h temporal pattern, with higher enzyme activity during the dark (activity) phase. It was found that stress can cause disruptions in biological circadian rhythms and in the cardiovascular system. Therefore, the aim of the present study was to examine the influence of acute stress exposure upon temporal patterns of NTPDase and 5-nucleotidase enzyme activities in rat blood serum.

**Methods:**

Adult male Wistar rats were divided into 4 groups: ZT0, ZT6, ZT12 and ZT18. Each group was subdivided in 4 groups: control, immediately, 6 h and 24 h after one hour of restraint stress. ATP, ADP and AMP hydrolysis were assayed in the serum.

**Results:**

All stressed groups showed significant decreases in all enzyme activities at ZT 12 and ZT 18 when compared with control.

**Conclusion:**

Acute stress provokes a decrease in nucleotidase activities dependent on the time that this stress occurs and this effect appears to persist for at least 24 hours. Stress can change levels of nucleotides, related to increased frequency of cardiovascular events during the activity phase. Altered levels of nucleotides in serum may be involved in cardiovascular events more frequent during the activity phase in mammals, and with their etiology linked to stress.

## Background

Extracellular adenosine 5'-triphosphate (ATP) and its breakdown products, adenosine 5'-diphosphate (ADP), adenosine 5'-monophosphate (AMP) and adenosine, can act as extracellular messengers in a range of biological processes through binding to purinergic receptors. In addition, they have been shown to have pronounced effects on a variety of biological processes such as neurotransmission, regulation of cardiac function and platelet aggregation [1], as well as pathological events including neurodegenerative and cardiovascular diseases [2]. ATP can be released via stimulation of sympathetic nerves [3] promoting vasoconstriction or vasodilatation, and contributes to platelet aggregation [4]. Additionally, its breakdown produces the nucleotide diphosphate (ADP), which is the most important platelet aggregator and also promotes vasoconstriction [5]. The nucleoside adenosine, also generated by ATP breakdown, is able to act as a vasodilator, inhibitor of platelet aggregation, and it may act as an endogenous cardioprotective substance [4].

Extracellular nucleotides can be hydrolyzed by a variety of enzymes that are located on the surface of cells, or by soluble forms in the interstitial medium or within body fluids [6]. Nucleoside 5'tri- and diphosphates (NTP and NDP) may be hydrolyzed by the nucleoside triphosphate diphosphohydrolase enzyme family (NTPDases), nucleotide phosphate inhibitor/phosphodiesterase family (NPP), alkaline phosphatases and ecto-5'-nucleotidase [6]. Eight different NTPDases have so far been described: NTPDases 1, 2, 3 and 8 are expressed on the cell surface with a catalytic site facing to the extracellular space, while NTPDases 4, 5 6, and are entirely intracellular [6]. The ecto-5'-nucleotidase is attached to the cell surface and may occur also in a soluble form via cleavage of its glycosyl-phosphatidylinositol (GPI)-anchor by phospholipase C [6]. These nucleotidases, together with 5'-nucleotidase, control the availability of ligands (ATP, ADP and adenosine) for both nucleotide and nucleoside receptors, and consequently, the duration and extent of receptor activation [4]. Previous work by our group demonstrated that ATPase and ADPase activities exhibit a 24-hour temporal pattern in rat blood serum, with activities increased during the dark period while AMPase activity did not display circadian variation [7].

Circadian organization is important to enable an organism to maintain equilibrium in response to the daily changes in the external environmental and prepare accordingly [8]. In mammals, a number of circadian patterns (~24-h) have been described, including the timing of endocrine hormone secretion (e.g. melatonin, corticosterone or cortisol, adrenocorticotropic hormone), body temperature, respiratory rate, heart rate, blood pressure and effect of drugs [9,10].

Some studies have demonstrated the influence of stress in modulating the purinergic system. Mild stress, such as a mild foot shock, is enough to promote specific changes in the hydrolysis of ATP and ADP in some tissues such as cerebral cortex [11]. Moreover, changes in the activities of enzymes involved in nucleotide hydrolysis have also been reported in spinal cord and blood serum after repeated restraint stress[12,13] and after acute restraint stress [14].

Considering that the temporal variation of nucleotidase activities [7] may be of great importance in regulating the cardiovascular system, and that acute stress disrupts both circadian rhythms [15,16] and the function of the cardiovascular system [17], we investigated the effects of acute restraint stress on the 24-h temporal pattern of ATPase, ADPase and AMPase enzyme activities in rat blood serum. More specifically, in this study we assessed the relationship between the time of day at which the stress is applied and the temporal course of the soluble nucleotidase activities after acute stress.

## Methods

### Animals

A total of 98 naive adult male Wistar rats (50-70 days old; 190-240 g weight) were used. The animals were maintained under a standard 12/12-h light/dark cycle [lights on at 07:00 h, Zeitgeber time (ZT) 0, and lights off at 19:00 h, ZT 12], in a controlled environment (22 ± 2°C), with rat chow and water ad libitum. The Zeitgeber time was used as a reference to detect the rhythmicity of the variables under study. The experimental protocol was approved by an Institutional Review Board for use in animals with procedures in accordance with the National Institutes of Health Guide for Care and Use of Laboratory Animals (Publication No. 85-23, revised 1985), the UK Animals Scientific Procedures Act 1986 and the European Community's Council Directive of 24 November 1986 (86/609/EEC).

### Experimental design

**Figure 1:**
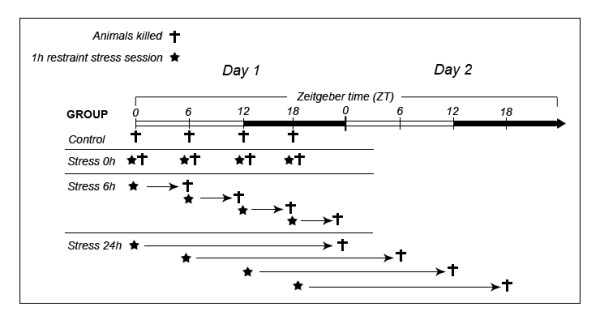
**Experimental design**.

Rats were habituated to the vivarium for 2 weeks before the beginning of the experiment. On the day of the experiment, the animals were divided into 4 groups according to time of day (ZT 0, ZT 6, ZT 12, and ZT 18) and each of these was subdivided into 4 groups according to time of death (control group, 0 hours, 6 hours, and 24 hours after acute stress) (see Figure 1). The rats were submitted to the model of acute restraint stress described by Torres et al. [12]. Trunk blood was drawn and blood samples were centrifuged in plastic tubes for 5 min at 5000 × g at room temperature [18]. Serum was obtained and frozen at -20°C until the enzyme assays were performed. To verify that enzymatic activity is not altered by freezing of the samples, we conducted a pre-study comparing frozen samples and samples obtained after decapitation of animals on the same day of the enzymatic assay. Blood samples were centrifuged in plastic tubes for 5 min at 3000× g at room temperature, the serum was separated, and it was used in the enzyme assay immediately (see below). We found no statistically significant difference between frozen and fresh samples.

### Enzyme assay

ATP, ADP, and AMP hydrolysis were analyzed using a modification of the method described by Oses and colleagues [19], differing only in number of duplicates, where we used triplicate number of samples. The reaction mixture, containing 0.5 to 1.0 mg serum protein in 112.5 mM Tris-HCl, pH 8.0, was preincubated for 10 min to equilibrate the mixture. The reaction was started by the addition of ATP or ADP (final concentration of 3.0 mM) and the mixture was incubated at 37°C in a final volume of 200 μl, for 40 min. The reaction was stopped by the addition of 200 μl 10% trichloroacetic acid (TCA). All samples were centrifuged at 5000 × g for 5 min to eliminate precipitated protein and the supernatant was used for the colorimetric assay. The inorganic phosphate (Pi) released was measured by the Malachite green method [20]. AMP hydrolysis was quantified essentially as described above for ATP and ADP hydrolysis. The reaction mixture, containing 3.0 mM AMP as substrate in 100 mM Tris-HCl, pH 7.5, was incubated with 0.5 to 1.0 mg serum protein at 37°C in a final volume of 200 μl. All other procedures were the same as described above for ATP and ADP hydrolysis. For all enzyme assays, incubation times, substrate and protein concentrations were chosen in order to ensure the linearity of the reactions. All samples were run in triplicate. In order to correct for non-enzymatic hydrolysis, we performed controls by adding the serum after the reaction was stopped with TCA. Enzyme activities were expressed as nmol of inorganic phosphate released per minute per milligram of protein (nmolPi/min/mg protein).

### Protein determination

Protein was measured by the Coomassie Blue method using bovine serum albumin as standard [21].

### Statistical analysis

Data were expressed as mean ± standard error of the mean (S.E.M.). Comparisons between groups were analyzed by one-way ANOVA followed by Tukey's test. Differences between groups were considered significant at *P *< 0.05. SPSS 17.0 for Windows was used for statistical analysis.

## Results

**Figure 2:**
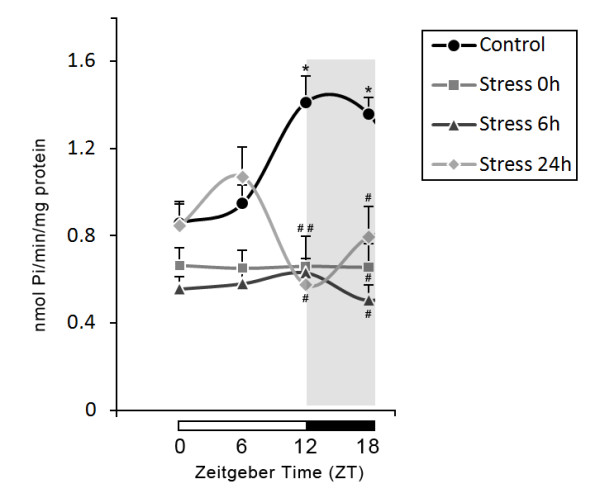
**Effects of stress on 24-h temporal pattern of ATPase activity in blood serum**. Values are expressed as mean ± S.E.M. specific activity (nmoles of Pi produced/min/mg protein). Number of animals per group = 5-10. * indicates significant differences (One-way ANOVA/Tukey, *P <*0.05) from ZT 0 and ZT 6 in control group. # indicates significant differences (One-way ANOVA/Tukey, *P <*0.05) from control group. Horizontal bars at the base of the graph represent day (white) and night (black) phase, with Zeitgeber times (ZTs) indicated below.

**Figure 3:**
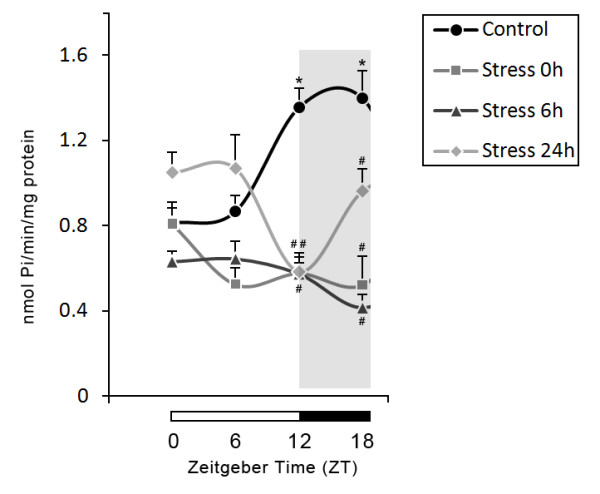
**Effects of stress on 24-h temporal pattern of ADPase activity in blood serum**. Values are expressed as mean ± S.E.M. specific activity (nmoles of Pi produced/min/mg protein). Number of animals per group = 5-10. * indicates significant differences (One-way ANOVA/Tukey, *P <*0.05) from ZT 0 and ZT 6 in control group. # indicates significant differences (One-way ANOVA/Tukey, *P <*0.05) from control group. Horizontal bars at the base of the graph represent day (white) and night (black) phase, with Zeitgeber times (ZTs) indicated below.

**Figure 4:**
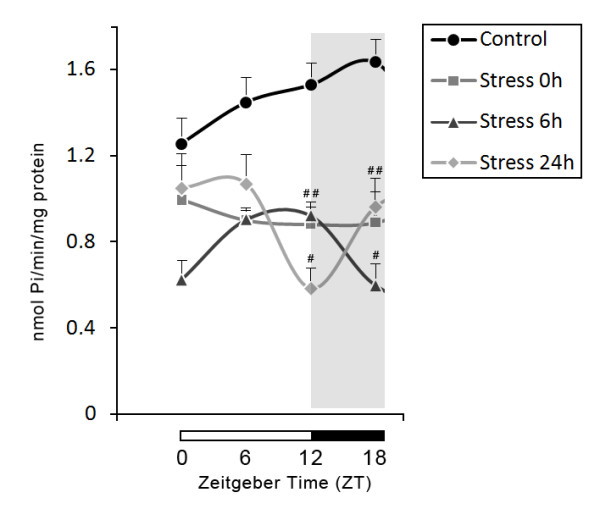
**Effects of stress on 24-h temporal pattern of AMPase activity in blood serum**. Values are expressed as mean ± S.E.M. specific activity (nmoles of Pi produced/min/mg protein). Number of animals per group = 5-10. # indicates significant differences (One-way ANOVA/Tukey, *P <*0.05) from control group. Horizontal bars at the base of the graph represent day (white) and night (black) phase, with Zeitgeber times (ZTs) indicated below.

A summary of all the results of stress effects upon temporal patterns of ATPase, ADPase and AMPase activities are presented in Figures 2, 3 and 4.

### Effects of stress on ATPase-ADPase activities in blood serum over 24-h

The hydrolysis of ATP and ADP were determined in blood serum at 0 h, 6 h and 24 h after the stress procedure during 24-hour at ZT 0, 6, 12, and 18. One-way ANOVA followed by Tukey's test revealed significant differences in the effect of stress on the 24-h profile of ATPase (F_15,82 _= 8.205, *P *< 0.001, Figure 2) and ADPase activities (F_15,82 _= 9.911, *P *< 0.001, Figure 3). In agreement with a previous study by our group, the control groups showed higher enzyme activities at ZT 12 and ZT 18 when compared to ZT 0 and ZT 6 [7]. All stressed groups showed a significant decrease in enzyme activities at ZT 12 and ZT 18 when compared with the control group (Figures 2 and 3).

### Effects of stress on AMPase activity in blood serum over 24-h

The hydrolysis of AMP was determined in blood serum at 0, 6 and 24 h after the stress procedure during 24-hour at ZT 0, 6, 12, and 18. One-way ANOVA followed by Tukey's test revealed significant differences in the effect of stress on the 24-h profile of AMPase activity (F_15,82 _= 7.361, *P *< 0.001, Figure 4). In agreement with a previous study by our group no difference was observed between the control groups at different ZTs. All stressed groups showed a significant decrease in enzyme activities at ZT 12 and ZT 18 when compared with the control group (Figure 4).

## Discussion

Our finding that ATPase activity and ADPase activity in blood serum were higher during the dark period (ZT 12 and ZT 18) than during the light period (ZT 0 and ZT 6) in control rats is consistent with a previous study in which we demonstrated that ATPase and ADPase activities (probably the NTPDase 1-like soluble enzyme) exhibit a 24-h temporal pattern [7]. In the present study, the activities of ATPase, ADPase and AMPase were decreased by acute (1 h) restraint stress during the dark period. We found that acute stress causes a loss of this temporal pattern in the nucleotidase activities, lasting up to 6 h and 24 h after the stressor event, although only when this occurred in the dark phase (ZT 12 and ZT 18). This suggests that the activities of nucleotidase enzymes are subject to a greater influence of acute stress during night hours than during daylight hours (possibly because enzymatic activity is already at its lowest during daylight hours), and this influence appears to persist for at least 24 hours. In addition, ATPase/ADPase ratio was the same in both groups (~1.0), demonstrating a parallelism between the two activities. This suggests the presence of soluble NTPDase 1-like enzyme in serum being affected by acute stress procedure. To our knowledge, this is the first investigation that verifies the effects of stress on the temporal pattern of serum nucleotidases, which are responsible for hydrolyzing adenine nucleotides.

This result is consistent with previous findings in humans [16] and in rodents [15] that showed the disruption of biological circadian rhythms by acute stress. Unpublished experiments performed by our group demonstrated that corticosterone, melatonin and glucose, which have well-defined circadian patterns in rat blood serum, are also affected by the acute stress procedure. Thus, it is possible to speculate that the disruption of the circadian timing of the activities of nucleotidase enzymes might explain, at least in part, the physiological process of the cardiovascular events that display a circadian pattern, such as blood pressure, heart rate, and vasodilatation [10]. Additionally, it could be suggested that the disruption of orchestrated activities of nucleotidase enzymes may be involved in the complex machinery of local oscillators in the heart, endothelium and vascular smooth muscle, as well as endocrine interactions and their regulation by feeding, stress and energetic demands [22]. In this context, stress may deregulate the circadian timing present in the cardiovascular system, and acute stress might trigger cardiovascular events including myocardial infarction, ventricular dysfunction and dysrhythmia [17].

Additionally, mechanisms involved in the acute stress response promote the critical secretion of glucocorticoids that is linked to the modulation of ATPase, ADPase and AMPase activities [14]. Here, it is important to emphasize that in our study the reduction in ATPase, ADPase and AMPase activities in blood serum caused by acute (1 h) restraint stress during night hours (corresponding to the period of wakefulness in humans) induced an increase in circulating levels of ATP, ADP, and AMP, and consequently decreased the production of adenosine. Therefore, the decrease of soluble enzyme (probably NTPDase 1-like) after stress can be related to increased release of hormones of stress like corticosterone. In the dark phase, corticosterone exhibits a temporal secretion profile with peak plasma levels occurring immediately prior to the onset of an organism's activity cycle [23]. However, we can not rule out a modulation of purinergic enzymes by the hormone melatonin that begins its release in the dark phase with peak plasma levels occurring in middle of the night [7].

It was verified that soluble nucleotidases seem to be co-released with the neurotransmitter ATP via stimulation of sympathetic nerves [3]. As is well known, ATP constricts vascular smooth muscle via the P2X receptor and together with norepinephrine stimulates platelet aggregation [4]. ADP, in turn, is a platelet aggregator and it promotes vasoconstriction via the P2Y12 receptor on vascular smooth muscle cells [5]. Together, ATP and ADP exert prothrombotic and proinflammatory effects. Moreover, in our results the AMPase activity decreased after stress during night hours with a consequent reduction in the production of adenosine. This could have important consequences for the cardioprotective functions of the latter mediators, such as vasodilatation induced via P1 receptors on smooth muscle, and inhibition of platelet aggregation [4].

Additionally, stress causes release of epinephrine from the adrenal glands, and norepinephrine is released together with ATP by the terminals of the sympathetic nervous system [24]. The elevation of norepinephrine and epinephrine in blood by itself can promote thrombosis through the vasoconstrictor properties of these mediators and a direct action on platelets via the α2a receptor [25]. In a similar way, under stress conditions ATP and ADP act in combination with epinephrine and norepinephrine and thus can contribute to cardiovascular events including thrombosis [4]. NTPDase 1-knockout mice exhibited disrupted development of the vasculature and presented hemostatic and thromboregulatory disturbances, and the recombinant soluble form of human NTPDase 1 was found to inhibit thrombosis [26]. Therefore, enzymes that degrade adenine nucleotides such as NTPDase 1 and 5'-nucleotidase are very important in the regulation of the cardiovascular system [6]. Accordingly, our results indicate a negative influence of acute stress upon nucleotidases (probably NTPDase 1-like and 5'-nucleotidase), while this form of stress appears to act directly on the hydrolysis of adenine nucleotides only during the night period in rats (day in humans) where there is a significant physiological increase of NTPDase 1 activity, suggesting a possible temporal deregulation of this enzyme [7]. Even though AMPase activity, possibly mediated by the 5'-nucleotidase enzyme, does not have a temporal pattern [7], the reduction in activity observed after acute stress only during the dark phase suggests a possible modulation of this enzyme.

In conclusion, acute stress decreased the hydrolysis of nucleotides in rat blood serum during the night (activity) phase, and this effect persisted for up to 24 hours. It is tempting to suggest that altered levels of nucleotides and nucleosides in serum may be involved in the more frequent occurrence of cardiovascular events during the activity phase in mammals [27], which has an etiology linked to stress [17].

## Competing interests

The authors declare that they have no competing interests.

## Authors' contributions

AS and BDC carried out the design of the study and performed the statistical analysis; LFM and JRR carried out the enzymatic assays and helped to shape the manuscript; WC, MPLH and AMOB participated in the design of the study; ILST coordinated the study, performed the statistical analysis, and helped to draft the manuscript. All authors read and approved the final manuscript.
